# Avaliação da Insuficiência Valvar Aórtica por Ecocardiografia: Conceitos Básicos e Novos

**DOI:** 10.36660/abc.20200559

**Published:** 2020-08-19

**Authors:** Said Alsidawi

**Affiliations:** 1 Minneapolis Heart Institute Abbott Northwestern Hospital Minneapolis Minnesota EUA Minneapolis Heart Institute at Abbott Northwestern Hospital, Minneapolis, Minnesota – EUA

**Keywords:** Insuficiência da Valva Aórtica, Regurgitação Aórtica, Velocidade do Fluxo Sanguíneo, Diagnóstico por Imagem/métodos, Ecocardiografia, Ecocardiografia Doppler, Ecocardiografia Tridimensional

O ecocardiograma continua sendo o padrão-ouro para o diagnóstico e classificação das cardiopatias valvares,^1^ apesar do avanço de outras modalidades de imagem. A insuficiência valvar aórtica (IA) é um distúrbio valvar comum^2^ que também pode ser um dos mais desafiadores para quantificar-se com precisão. O ecocardiograma ajuda a avaliar a gravidade da IA utilizando várias técnicas bidimensionais, tridimensionais e de Doppler colorido, mas o mais importante é que oferece uma oportunidade única para a avaliação hemodinâmica, que é extremamente importante na classificação da gravidade da IA.

No artigo intitulado: “Integral velocidade-tempo da regurgitação aórtica: um novo marcador ecocardiográfico na avaliação da gravidade da insuficiência aórtica”,^3^ os autores testaram, como prova de conceito, a correlação entre a integral velocidade-tempo (IVT) da IA e a gravidade da insuficiência aórtica em uma análise multivariada e mostraram a relação inversa entre a IVT da IA e a gravidade da IA, independente do diâmetro e volume do ventrículo esquerdo, frequência cardíaca, pressão arterial diastólica ou fração de ejeção do ventrículo esquerdo. Eles também mostraram que a IVT da IA é um método que pode ser facilmente obtido e reproduzido para avaliar a gravidade da IA, em comparação com outros métodos comumente utilizados, como a Área de Superfície da Isovelocidade Proximal (PISA). Este estudo apresenta um conceito interessante e promissor que aumentará o nível de confiança ao avaliar a gravidade da IA. Também faz sentido fisiologicamente, pois os pacientes com IA grave têm um gradiente diastólico menor entre a aorta e o ventrículo esquerdo (pressão diastólica final mais alta do ventrículo esquerdo e pressão arterial diastólica mais baixa) que teoricamente resultarão em um valor menor de IVT da IA devido à rápida equalização pressão entre a aorta e o ventrículo esquerdo.

Vale ressaltar, porém, que existem algumas limitações para este estudo. Primeiro, há uma falta de padrão ouro para a avaliação da gravidade da IA, além da “opinião de especialistas”. Segundo, o grupo com IA grave é provavelmente composto de dois grupos separados: o grupo IA grave aguda e o grupo IA grave crônica. É importante distinguir esses dois grupos, pois a IA grave, bem compensada e crônica é provavelmente hemodinamicamente semelhante à IA moderada, com um valor de IVT da IA maior do que aqueles com IA grave aguda. A falta de associação entre a gravidade da IA e o meio-tempo de pressão neste estudo confirma que o grupo com IA grave é provavelmente uma mistura de pacientes com cronicidade variável. Terceiro, alguns jatos de IA serão muito desafiadores para obter uma amostra por Doppler de onda contínua, dada sua excentricidade. Isso é principalmente significante em pacientes com válvulas aórticas bicúspides ou unicúspides que tendem a ter jatos regurgitantes muito excêntricos. Por fim, será necessário um acompanhamento clínico para avaliar a sobrevida, a necessidade de cirurgia valvar aórtica ou outros eventos adversos com base no valor da IVT da IA para verificar a utilidade do conceito.

## Conclusão

A introdução de novos conceitos ou técnicas que ajudam na classificação da gravidade da IA é um recurso valioso. A IVT da IA é um conceito promissor que é fisiologicamente correto e parece ser reproduzível. Serão necessários ensaios clínicos maiores para avaliar melhor seu papel e, mais importante, seu valor prognóstico e correlação com os resultados clínicos.

É indispensável, no entanto, ter em mente que é muito improvável que encontremos um marcador ecocardiográfico único que seja um padrão ouro na avaliação da gravidade da IA. Os ecocardiografistas devem manter a mente aberta e integrar todos os dados disponíveis para chegar a uma conclusão final. Isso inclui o seguinte:^1^

1. Dados clínicos (pressão de pulso ampla, frequência cardíaca, sintomas)
2. Avaliação bidimensional e tridimensional da válvula aórtica (avaliação da anatomia valvar quanto ao número de folhetos, perfurações, vegetação, prolapso da cúspide etc.) e câmaras cardíacas (tamanho e função do VE e VR, tamanho do AE)
3. Doppler colorido (Vena Contracta,^4^ largura do jato comparado à largura da VSVE, avaliação PISA^5^ quando possível e quantificação 3D com Doppler colorido^6^ )
4. Doppler espectral (densidade do sinal do jato, meio-tempo de pressão, IVT da IA, IVT da VSVE, IVT valvar aórtica, padrão de influxo mitral, estimativa da pressão sistólica do ventrículo direito, etc.).

Entretanto, como em outras lesões valvares, ecocardiografistas e estagiários devem evitar diagnosticar a gravidade da IA com base apenas no Doppler colorido, mesmo que seja tentador fazê-lo inicialmente. A avaliação das consequências hemodinâmicas da IA deve ser um componente-chave da avaliação. Por exemplo, é improvável que o diagnóstico de IA grave com tamanho diastólico final normal do ventrículo esquerdo, sem reversão do fluxo diastólico na aorta torácica descendente ou abdominal ou com uma pressão de pulso normal seja preciso e deve ser reavaliado.

Além disso, a utilização de outras modalidades para avaliar a gravidade da IA pode ser necessária quando os dados ecocardiográficos e clínicos forem inconclusivos ou contraditórios. A ressonância magnética cardíaca (RMC) tem um papel importante e promissor na avaliação da gravidade da IA, especialmente com jatos excêntricos ou vazamentos valvares periprotéticos. Ajuda a avaliar a fração regurgitante, utilizando imagens de contraste de fase e o tamanho e a função dos ventrículos esquerdo e direito com boa precisão.^7^ A tomografia computadorizada (TC) cardíaca também pode ser útil para identificar vazamentos periprotéticos e orientar procedimentos cirúrgicos e percutâneos.^8^ Finalmente, um aortograma bem executado, realizado no laboratório de cateterização, pode ser muito valioso quando outras modalidades de teste são inconclusivas.
