# Supplementary material for: Avaliação da Insuficiência Valvar Aórtica por Ecocardiografia: Conceitos Básicos e Novos
Source: Arq Bras Cardiol. 2020 Aug 19;115(2):261–2. [Article in Portuguese] doi: 10.36660/abc.20200559 (PMC8384290; doi:10.36660/abc.20200559)
Supplement: Translation

# Translation: Assessment of Aortic Valve Regurgitation by Echocardiography: Basic and New Concepts

**Authors:** Said Alsidawi

**Affiliations:** 1 USA Minneapolis Heart Institute at Abbott Northwestern Hospital,Minneapolis, Minnesota – USA

**Keywords:** Aortic Valve Insufficiency, Aortic Regurgitation, Blood Fow Velocity, Diagnostic, Imaging, Echocardiography, Echocardiography, Doppler, Echocardiography, Three-Dimensional

Echocardiography remains the gold standard for the diagnosis and grading of valvular heart diseases,^1^ despite the development of other imaging modalities. Aortic valve regurgitation (AR) is a common valvular disorder,^2^ which can also be one of the most challenging to accurately quantify. Echocardiography helps in assessing the severity of AR utilizing multiple two-dimensional, three-dimensional and color Doppler techniques, but most importantly, offers a unique opportunity for hemodynamic evaluation, which is extremely important when grading the severity of AR.

In the article entitled: “Velocity-time integral of aortic regurgitation: a novel echocardiographic marker in the evaluation of aortic regurgitation severity”, the authors tested, as a proof of concept, the correlation between AR velocity-time integral (VTI) and the severity of aortic valve regurgitation in a multivariate analysis and showed the inverse correlation between AR VTI and AR severity, regardless of left ventricular diameter, volume, heart rate, diastolic blood pressure or left ventricular ejection fraction. They also showed that AR VTI is an easily obtainable and reproducible method to assess AR severity when compared to other commonly used methods, such as Proximal Isovelocity Surface Area (PISA). This study introduces an interesting and promising concept that will add to confidence level when assessing AR severity. It also makes physiological sense, as patients with severe AR will have a smaller diastolic gradient between the aorta and the left ventricle (higher left ventricular end diastolic pressure and lower diastolic blood pressure), which will theoretically result in a smaller AR VTI value due to rapid equalization of pressure between the aorta and the left ventricle.

It is worth noting, however, that there are a few limitations to this study. First, there is a lack of a gold standard for the assessment of AR severity other than an “expert opinion”. Second, the severe AR group is probably consisted of two separate groups: the acute severe AR group and the chronic severe AR group. It is important to distinguish between these two groups as chronic, well compensated, severe AR is likely to be hemodynamically similar to moderate AR with a larger AR VTI value, when compared to those with acute severe AR. The lack of association between AR severity and pressure half time in this study supports the fact that the severe AR group is probably a mix of patients with variable chronicity. Third, some AR jets will be very challenging to sample by continuous wave Doppler, given their eccentricity. This is mainly notable in patients with bicuspid or unicuspid aortic valves, which tend to have very eccentric regurgitant jets. Finally, a clinical follow-up to assess survival, need for aortic valve surgery or other adverse events based of AR VTI value will be needed to verify the utility of the concept.

## Conclusion

Introducing new concepts or techniques that can help grading AR severity is a valuable resource. AR VTI is a promising concept that is physiologically sound and appears reproducible. Larger clinical trials will be needed to further assess its role and, more importantly, its prognostic value and correlation with clinical outcomes.

It is imperative, however, to keep in mind that it is very unlikely that we will find a single echocardiographic marker that will be a gold standard when assessing AR severity. The echocardiographist has to keep an open mind and integrate all available data to come to a final conclusion. This includes the following:^1^

1. Clinical data (wide pulse pressure, heart rate, symptoms)
2. Two-dimensional and three-dimensional evaluation of the aortic valve (valve anatomy assessing for number of leaflets, perforations, vegetation, cusp prolapse etc...) and cardiac chambers (LV and RV size and function, LA size)
3. Color Doppler (Vena Contracta,^4^ jet width compared to LVOT width, PISA^5^ evaluation when feasible and 3D color Doppler quantification^6^ )
4. Spectral Doppler (Jet signal density, pressure half time, AR VTI, LVOT VTI, aortic valve VTI, mitral inflow pattern, right ventricular systolic pressure estimation etc…).

However, as with other valve lesions, echocardiographists and trainees should refrain from diagnosing AR severity based on color Doppler alone, even if it is tempting to do so initially. Assessing the hemodynamic consequences of AR should be a key component of the evaluation. For example, diagnosing severe AR in the setting of a normal left ventricular end diastolic size, without diastolic flow reversal in the descending thoracic or abdominal aorta, or with a normal pulse pressure is unlikely to be accurate and should be reassessed.

Furthermore, utilizing other modalities to assess AR severity might be needed when echocardiographic and clinical data are inconclusive or contradictory. Cardiac Magnetic Resonance Imaging (MRI) has an important and promising role in assessing AR severity, especially with eccentric jets or peri-prosthetic valvular leaks. It helps assess the regurgitant fraction utilizing phase-contrast imaging and left and right ventricular size and function with good accuracy.^7^ Cardiac Computed Tomography (CT) can also be helpful to identify peri-prosthetic leaks and guide surgical and percutaneous procedures.^8^ Finally, a well-performed aortogram carried out in the catheterization laboratory can be very valuable, when other testing modalities are inconclusive.
